# Refinement of RNA Structures Using Amber Force Fields

**DOI:** 10.3390/cryst11070771

**Published:** 2021-07-01

**Authors:** Jonathon G. Gray, David A. Case

**Affiliations:** Department of Chemistry and Chemical Biology, Rutgers University, Piscataway, NJ 08854, USA

**Keywords:** crystallographic refinement, force fields, RNA

## Abstract

Atomic models for nucleic acids derived from X-ray diffraction data at low resolution provide much useful information, but the observed scattering intensities can be fit with models that can differ in structural detail. Tradtional geometric restraints favor models that have bond length and angle terms derived from small molecule crystal structures. Here we explore replacing these restraints with energy gradients derived from force fields, including recently developed integral equation models to account for the effects of water molecules and ions that are not part of the explicit model. We compare conventional and force-field based refinements for 22 RNA crystals, ranging in resolution from 1.1 to 3.6 Å. As expected, it can be important to account for solvent screening of charge–charge interactions, especially in the crowded environment of a nucleic acid crystal. The newly refined models can show improvements in torsion angles and hydrogen-bonding interactions, and can significantly reduce unfavorable atomic clashes, while maintaining or improving agreement with observed scattering intensities.

## 1. Introduction

RNA ribonucleic acid is involved in a wide variety of cellular processes, and atomic models describing their structures (often derived from X-ray diffraction studies) can be a key element in understanding their functions. At resolutions beyond about 2.5 Å, there can be many models that fit the diffraction data almost equally well. In spite of the use of restraints on bond lengths and angles, there is a persistence of geometric outliers in RNA models developed from crystallographic data [1-3]. These outliers not only arise due to low resolution in many RNA structures [1,4], but also due to the use of geometric restraints that do not consider the full range of electrostatic interactions and hydrogen-bonding terms that can be important for structural optimization [5].

There have been a variety of attempts to go beyond conventional bond and angle restraints for RNA refinement. The phenix refinement method has added nucleic acid secondary structure restraints to maintain proper base pair hydrogen bonding, planarity, and stacking [6]. These, however, do not include an explicit energetic term, and enforce hydrogen bonding through restraint parameters that have to be specified in advance. In an attempt to get better structural models, the ERRASER project [1] used the Rosetta energy score function to guide real space refinement of the structure, individually modifying outlier bonds, angles, etc., to improve the overall structure, followed by a final step of reciprocal space refinement within phenix.

There is also a long history of efforts of combine X-ray and force-field based restraints. Some of the earliest implementations of refinement programs employed molecular mechanics force fields [7,8], and this idea has been continued in recent years, including the use of quantum mechanics as well as force fields [9-14]. The phenix refinement program [15] now includes an interface to Amber codes, allowing the use of gradients from force fields to replace more conventional bond and angle restraints derived from small molecules [11]. This interface has been shown to guide reciprocal space refinement of protein structures with considerable improvement over conventional refinement, especially at lower resolution. Here we explore the use of this interface on RNA structure refinement, incorporating recently developed methods to account for solvent molecules (ions and water) that are not explicitly represented in the atomic models.

## 2. Materials and Methods

### 2.1. Structures Examined

In order to have a wide-ranging set of RNA structures, we started with the set of structures used to test ERRASER [1]. After removing structures that were difficult to build due to size or difficult to parameterize units (ribosomal subunits, osmium-ion-containing structure, other building issues) and adding in three structures of an RNA mimic of the sarcin/ricin domain of the 23S–28S ribosomal RNA, we arrived at a set of 22 structures ranging in resolution from 1.04 to 3.6 Å. The set includes riboswitches, pseudoknots, ribozymes, and viral RNA domains, and some of the structures include the U1 small nuclear ribonucleoprotein A. For a description of each structure, see Table 1.

### 2.2. Refinements Using Phenix

Our main set of refinements used functionality built into phenix.refine. For standard runs (called “cdl”) we used the default conformation-dependent library, running 10 macrocycles of coordinate and isotropic ADP refinement, starting from the deposited structure, and using the maximum likelihood (“ml”) target [20,21]. Amber refinements started with the phenix.AmberPrep command to prepare the system, and again with 10 macrocycles of refinement using the use_amber=True flag. Other refinement parameters used the defaults in phenix. At these resolutions, this implies that only isotropic B-factors were refined, and that hydrogen atoms did not contribute to the calculated scattering factors.

For cdl refinements, we used the default weighting between X-ray and geometric restraints, based on heuristics that involve the gradients of each term. For amber refinements, we fixed the relative weights in the following fashion: The “ml” target is defined as the negative of the logarithm of the likelihood function for the observed structure factors. If one assumes a Boltzmann distribution for the force field likelihood, its negative log would be *E_MM_/k_B_T*, where *T* is some effective temperature. We provisionally take this as ambient temperature where the crystals were grown, even if these were later cyro-cooled. Adding the ml target and *E_MM_/k_B_T* gives the total target function, effectively assigning a “theoretical” relative weight. While this is an attractive idea, it is not clear that such a fixed relative weight is really optimal, and future studies will explore other possible combinations.

### 2.3. Refinements Using 3D-RISM

Integral equation models for solvation have been used for some time to describe both the distribution of waters and ions near biomolecules, as well as to describe the thermodynamic effects arising from the perturbation of the solvent when a solute (RNA here) is introduced [22-25]. Table 2 gives the parameters used. Grid spacings were chosen so that execution times would be within a reasonable range; no calculation took more than 6 min on 16 cores of a single Intel 6230 CPU. Most simulations somewhat arbitrarily used a 100 mM NaCl water solvent, but in some cases we attempted to mimic the divalent ion concentrations in cells, or in the crystal mother liquor (for 2oiu). A discussion of the computed effects of solvent ion concentrations is given elsewhere [26], but these are generally small.

We used codes in AmberTools [25] to carry out the coordinate refinements, and phenix.refine for isotropic B-factor refinements [15], alternating cycles of 150 refinement steps of coordinate refinement with five macro-cycles of B-factor optimization. Both coordinate and B-factor refinements used the default maximum-likelihood target from phenix.refine. Starting structures were taken from the PDB deposition.

## 3. Results

Two main sets of results that are presented here. The first uses the Amber force field to create geometric restraints in phenix, and to use these for refinement of 22 RNA crystal structures of varying resolution. This is essentially an extension of earlier work [11] that explored the use of this method in protein structures. The approach, as with proteins, is to carry out parallel sets of refinements, comparing conventional bond and angle restraints and a force-field approach where gradients of an energy function are added to those arising from an X-ray term that describes the level of agreement between observed and modeled structure factors.

The principal feature that differs between nucleic acids an proteins is the high charge density due to phosphate groups, and the need to describe the screening of such electrostatic interactions by water and ions that make up the bulk solvent. This led us to a second set of results, using a newly developed periodic version of the 3D-RISM integral equation model. We applied this approach for seven of the lower resolution structures, where details of the atomic positions are less well determined by the X-ray data alone.

### 3.1. Refinements Using Standard Phenix Approaches

**Figure 1.**
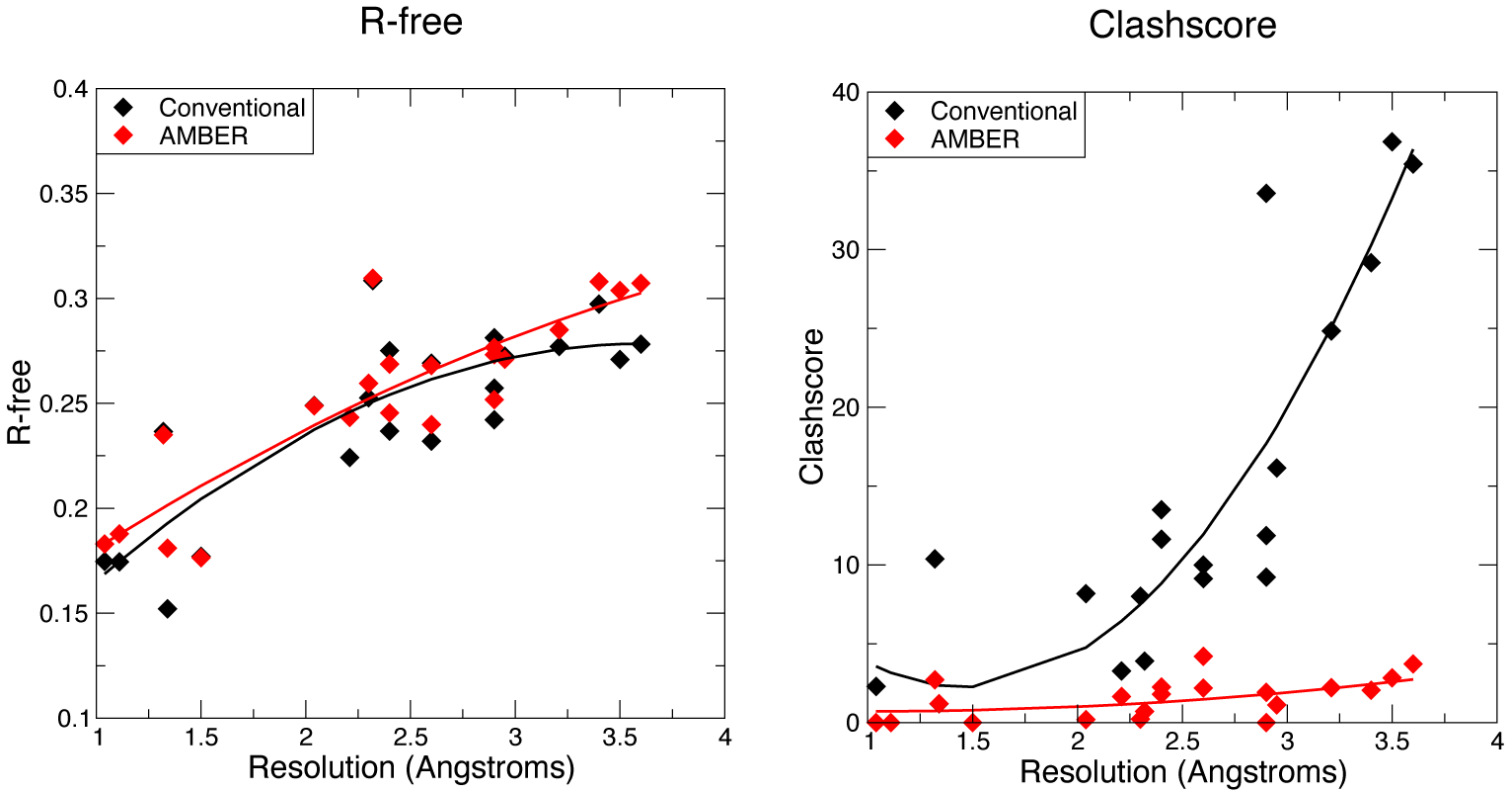
Values for R-free and Molprobity clashscore for the AMBER-restrained and conventionally restrained refinements.

**Figure 2.**
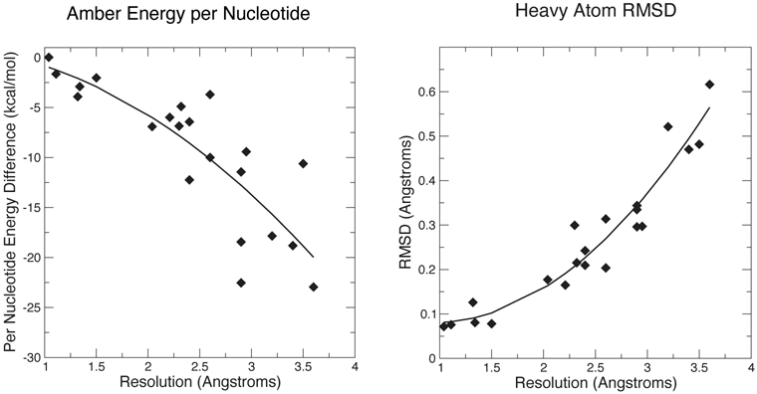
Energies and structural deviations for Amber and conventional refinements. The left panel shows the difference in Amber energy (per nucleotide) between Amber and conventional refinements; negative values indicate that the Amber refinement has a lower energy. The Amber energy estimate includes the solvation free energy estimated from 3D-RISM.

Figures 1 and 2 show some key points of the comparison between conventional and force-field based restraints when applied to RNA crystal structures; more details are provided elsewhere [26]. The results are much in accord with experience with proteins: At resolutions below 2 Å, the method of refinement makes little difference, since the density is clear enough to place most atoms in a good location. At lower resolutions, use of a force field can dramatically lower the incidence of bad clashes, and the structural difference between conventional and force-field geometric restraints increases as the resolution becomes poorer. Generally, the agreement with X-ray is little–changed. As seen in Figure 2, the Amber energy per nucleotide (compared to a conventional refinement) is nearly the same at high resolution, but gets progressively better at lower resolutions. This is, of course, expected, since the force field energy is being optimized in the Amber refinements. The Amber RNA force field is far from perfect, but a large number of studies suggest that better energies are indeed generally correlated with better structures [27].

Sugar puckers are important determinants of RNA strucure, and are generally very similar in the conventional and force-field based refinements. Tables 3 and 4 (below) show pucker outliers reported by Molprobity. The fractions of unusual sugar puckers are small, sometimes slightly favoring one method and sometimes the other. As we have noted earlier [11], outliers can be indicators of problems with local geometry that are worthy of investigation.

**Figure 3.**
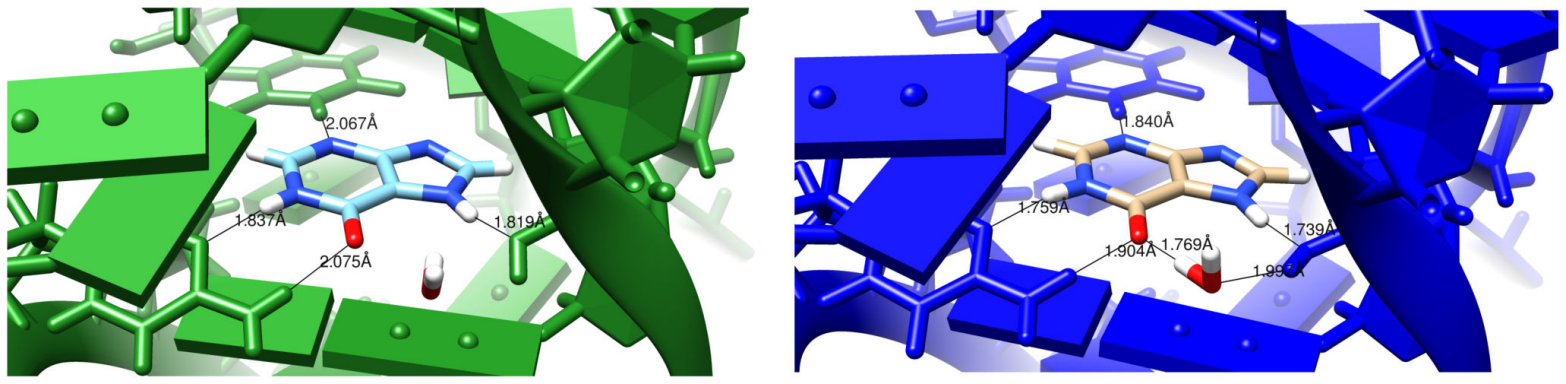
Images comparing conventionally and AMBER-restrained (green and blue, respectively) refinement outputs for 4fe5 [28]. The difference here is in distances and also the additional hydrogen bonds involving a nearby solvent molecule in the AMBER image. The ligand being examined is hypoxanthine (HPA).

It should be emphasized that the structural changes upon refinement are not large (Figure 2), with the average shift always less than 0.6 Å. Such changes may or may not reveal interesting structural details. Figure 3 shows an example where the details of an RNA–ligand interaction are clarified, even in a high-resolution (1.3 Å) structure. The details of hydrogen bonding interactions, including a water-mediated contact between the RNA and ligand, are much clearer in the force-field refined model.

**Figure 4.**
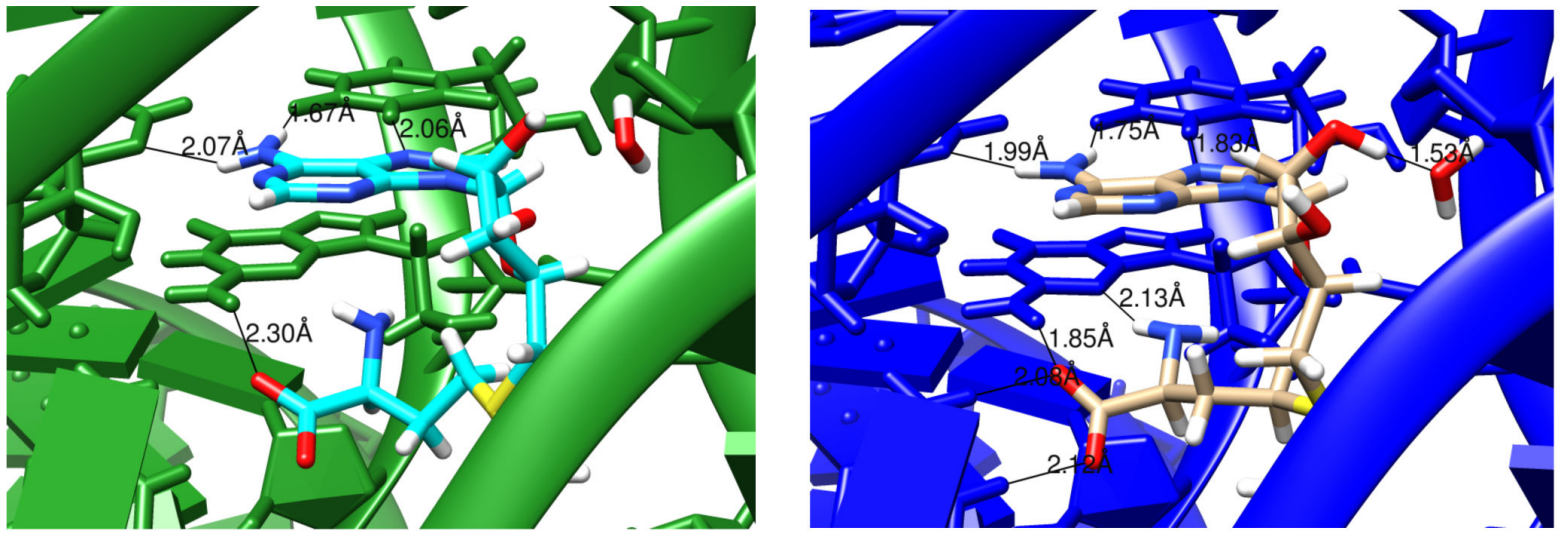
Images comparing conventionally and AMBER-restrained (green and blue, respectively) refinement outputs for 2gis [29]. Hydrogen bonds indentified by the cpptraj module in AmberTools are marked with the distances involved. The ligand being examined is S-adenosylmethionie (SAM).

Similar behavior is seen in Figure 4 for a lower-resolution (2.9 Å) model of a SAM riboswitch, pdb code 2gis [29]. Here the aromatic part of SAM is intercalated between U57 (above) and G58 (below). In the conventional refinement, only four hydrogen bonds are modeled between the ligand and RNA, whereas eight hydrogen-bonding contacts are present in the Amber-refined structure. Of course, one should not accept the latter model uncritically, but the interactions make good sense, and the resulting model is fully consistent with the X-ray diffraction data.

### 3.2. Refinements Using an Integral Equation Solvent Model

**Figure 5.**
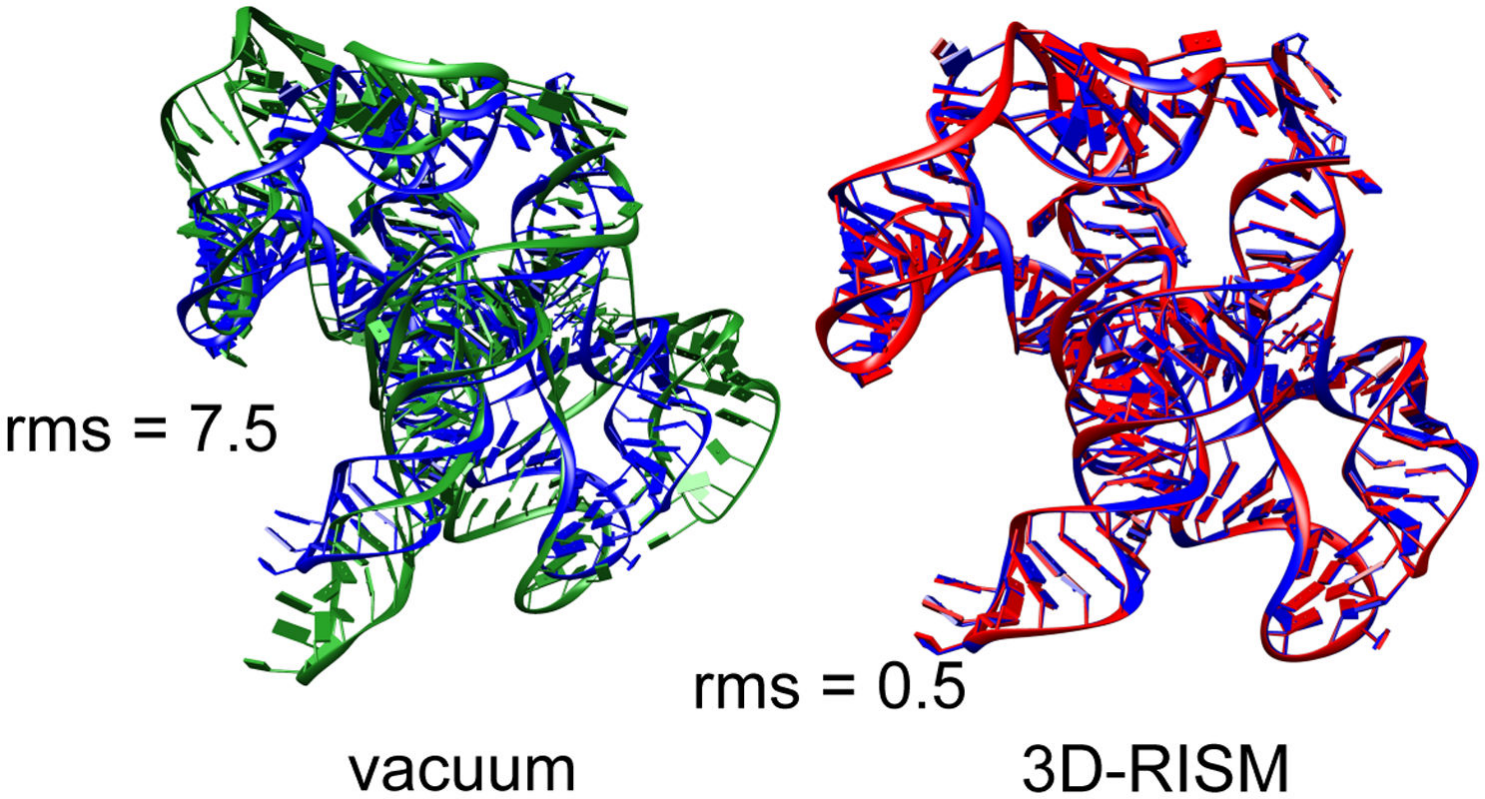
Blue: Experimental structure from X-ray crystallography (PDB ID 1y0q); red: Structure from a 3D-RISM crystal minimization; green: Structure from a crystal minimization with no solvent correction. RMS give the root-mean-square deviation (in Å) of all non-hydrogen atoms from the crystal structure.

The need to include the energetic aspects of solvation is especially important for nucleic acids crystals, where there are many charged phosphate groups in close proximity, and generally only a small number of counter ions are visible in the electron density maps. As an example, we consider the group I intron product complex (PDB code 1y0q [16]). Figure 5 shows results of minimization calculations in the crystal lattice, with and without the 3D-RISM implicit solvent model. The system (60,288 solute atoms) was minimized for 400 steps of conjugate gradient minimization, with a final RMS gradient of 0.02 kcal/mol. Here each RISM energy evaluation required 9 min of time on 16 threads on a single CPU. (Smaller systems can be considerably faster.)

It is clear that the lack of solvent screening of the phosphate-phosphate interactions in the “vacuum” minimizations results in an expansion of the system, even within the constraints of the crystal lattice, whereas the 3D-RISM calculations show excellent fidelity to the experimental structural models. In a refinement calculation without the implicit solvent model, the force-field energies would be fighting against the X-ray restraints, whereas the results in Figure 5 suggest that this would be much less true if 3D-RISM were employed.

Table 3 shows results for several crystallographic refinement calculations for the group I intron, pdb code 1y0q [16]. The diffraction data here are only at 3.6 Å resolution, so many structural details are not well-determined by the X-ray data alone. The first column shows the deposited results and gives statistics from the molprobity program [30]. The next two columns show parallel refinements (starting from the deposited structure) using phenix: The “phenix_cdl” column uses the default geometric restraints from its Conformational Dependent Library, which are largely similar to conventional Engh-Huber restraints. The “phenix_amber” column replaces the cdl restraints with forces from the Amber force field, as described elsewhere [11]. This force field model has no implicit solvent contribution, and hence no charge-screening effects. The final column adds in the 3D-RISM model.

The first row gives an overall score as assigned by Molprobity, where lower values are better; the score is set such that its value, averaged over many PDB entries, roughly matches the resolution. In this case, scores for the final two columns, where force field restraints are used, are significantly below the actual resolution of the data. The next four rows show scores for bad atomic clashes, for sugar puckers that are outside the range seen in high-resolution structures, and for the extent to which RNA torsional angles match those seen in high-resolution structures. For the clashscore and outliers, a lower number is better, whereas for the “suiteness”, which reflects the extent to which groups of torsion angles for an individual nucleotide resemble those expected, a higher values is better. The R-work and R-free rows measure the agreement with observed X-ray scattering amplitudes.

The results shown here are representative, as shown in Table 4, which gives 3D-RISM results for six additional low-resolution structures. The molprobity and clash scores are significantly improved when a force field is used, and torsion-angle scores improve to a smaller extent. These improvements are not made at the expense of X-ray constraints. Averaged over the seven structures in Tables 3 and 4, the overall molprobity score decreases from 2.83 to 1.77, and the clashscore from 22.5 to 0.8. The torsion angle statistics also improve, although to a less dramatic extent, with the average suiteness increasing from 0.462 to 0.528. These structural changes are accompanied by a decrease in average R-free from 0.277 to 0.255.

## 4. Discussion

The results presented here illustrate choices that can be made at the final stages of crystallographic refinement, in an attempt to improve physical realisism of the final atomic model, while still maintaining (or improving) the agreement with observed scattering factors. A typical use case would be to adopt these ideas as a final step, once conventional refinment is complete. The new and old atomic models can then be tested in a variety of ways to learn about their relative strengths and weaknesses. Considerable experience is available for proteins [11], showing that improvements are generally (but not always) obtained when force fields are used, and that a force-field refinement may pinpoint localized problems in atomic models that are not always easy to spot in other ways.

Here we report preliminary results that extend this idea to RNA. Nucleic acids have more torsional flexibility along the backbone than do proteins, but have fewer challenges in the modeling of sidechains (bases). Results for the Amber-based refinement in phenix suggest that benefits seen in proteins carry over to RNA, but that neglect of charge–charge screening by water and ions in the bulk solvent is a shortcoming worth addressing. One option is to use 3D-RISM theory to model the effects of solvent. While expensive, this option appears to give good results for seven low-resolution RNA data sets we tested. Even though the structural changes are small, they can clarify structural details like hydrogen bonding and stacking, as shown in Figures 3 and 4.

It should be be emphasized that the results presented here represent a proof of principle, and not a systematic study of best practices. There are a number of areas where somewhat arbitrary choices were made for the calculations presented here, and which are probably not optimal:

1. The best relative weight of X-ray and geometric restraints is always a thorny issue in refinement. Heuristic approaches are often used, which are based on experience with particular types of geometric restraints. An alternate idea, used here, is to assume that the most likely prior distribution (in the absence of X-ray data) assumes a Boltzmann form with some effective temperature. This can be combined with a maximum likelihood estimate for the X-ray terms to establish the relative weighting. Further study of these alternatives is warranted, although in practice various relative weights often give quite similar results.
2. We have not optimized the parameters involved in integral equation models for bulk solvent. There is flexibility in choosing the solvent composition, the grid spacing, and the closure relations needed for these models [22-24]. Beyond this, entirely different approaches to incorporating bulk solvent electrostatics exist, including Poisson-Boltzmann and generalized Born models [31]. These alternatives are not presently implemented for compact periodic systems like macromolecular crystals, but this possibility might be worth exploring.
3. The coordinate updates used here were restricted to minimization, in our case starting from the deposited atomic model. This may well end up with atomic models that are trapped in local minima. More robust procedures, such as simulated annealing, should be explored to increase the radius of convergence from what might be an imperfect starting structure. Such extensions might make it useful to apply force-field restraints at earlier stages of refinement.
4. The calculations used here assume that the entire solvent can be described by the correlation functions arising from the 3D-RISM model. In practice, some “localized” waters or ions can often be identified from electron density maps. In such cases, a mixed model that includes some explicit waters and ions could be combined with an integral equation model for what is left. Figuring out the optimal combination will depend a lot on resolution and the nature of the solvent channels in any particular crystal.

The general approach described here should also be useful for building atomic models into cryoEM maps [32]. One advantage is that much less expensive implicit solvent models (such as generalized Born theory [31]) can be used for non-periodic models. We will report explorations in this area in a separate publication.

## Figures and Tables

**Table 1.** Structures considered here. “U1” indicates the presence of the small nuclear protein U1. Structures in bold were also refined using a 3D-RISM implicit solvent model.

PDB ID	Description	Resolution
1q9a	sarcin/ricin domain from *E.coli* 23S rRNA	1.04
**1y0q**	active group I ribozyme-product complex [16]	3.60
**2a43**	luteoviral pseudoknot [17]	1.34
2gdi	thiamine pyrophosphate-specific riboswitch	2.04
2gis	SAM riboswitch mRNA regulatory element	2.90
**2oiu**	L1 ribozyme ligase circular adduct [18]	2.60
2pn3	Hep C IRES subdomain 2a	2.90
2pn4	Hep C IRES subdomain 2a	2.32
2qus	hammerhead G12A mutant pre-cleavage	2.40
2ygh	SAM-I riboswitch, with S-adenosylmethionine	2.60
**3bo3**	group I intron (U1) [19]	3.40
**3e5e**	SMK box (SAM-III) riboswitch with SAH	2.90
**3f2q**	FMN riboswitch with FMN	2.95
3gx5	T. tencongensis SAM-I riboswitch	2.40
**3iwn**	bacterial c-di-GMP riboswitch (U1)	3.20
3mxh	c-di-GMP riboswitch from V. cholerae (U1)	2.30
**3r4f**	prohead RNA	3.50
3tzr	riboswitch complex from Hep C IRES	2.21
480d	sarcin/ricin domain from *E. coli* 23S rRNA	1.50
483d	sarcin/ricin domain from *E. coli* 23S rRNA	1.11
483d	sarcin/ricin domain from *E. coli* 23S rRNA	1.11
**4fe5**	xpt-pbuX guanine riboswitch aptamer domain	1.32

**Table 2.** 3D-RISM parameters. Grid spacing in Å.

PDB ID	Spacing	Grid Size	Solvent
1y0q	1.0	96 × 160 × 224	0.1 M NaCl
2oiu	0.5	96 × 224 × 144	0.1 M MgCl_2_, 1.29 M NaCl
3bo3	1.0	112 × 112 × 256	0.1 M NaCl
3e5e	1.0	100 × 112 × 96	0.02 M MgCl_2_, 0.14 M KCl
3f2q	0.8	90 × 96 × 192	0.1 M NaCl
3iwn	1.0	32 × 96 × 188	0.1 M NaCl
3r4f	1.0	108 × 112 × 224	0.1 M NaCl
4fe5	0.8	168 × 48 × 64	0.02 M MgCl_2_, 0.14 M KCl

**Table 3.** Results for several test refinements of 1y0q. The first seven rows come from the molprobity program [30]; the root-mean-square (RMS) change from the deposited structure is computed for all non-hydrogen atoms.

	1y0q	Phenix_cdl	Phenix-Amber	3D-RISM
molprobity score	3.35	3.18	2.31	1.91
clashscore	53.7	35.4	3.7	0.9
pucker outliers (%)	8.6	8.6	10.7	8.2
suite outliers (%)	22.5	27.0	27.5	18.9
average suiteness	0.492	0.414	0.307	0.557
R-work	0.277	0.221	0.264	0.251
R-free	0.310	0.278	0.307	0.293
RMS from deposited	0.00	0.36	0.71	0.37

**Table 4.** Comparison of deposited and 3D-RISM-refined structures. Outliers are in percent; RMS vs deposited is in Å, averaged over all non-heavy atoms.

Resolution, Å	2oiu 2.6	RISM	3r4f 3.5	RISM	3e5e 2.9	RISM	3f2q 2.95	RISM	3iwn 3.2	RISM	3bo3 3.4	RISM
molprobity score	2.59	1.73	3.21	1.65	2.22	2.07	2.50	1.84	3.63	1.59	2.33	1.59
clashscore	8.0	0.2	37.8	0.0	2.8	1.7	6.4	0.6	42.6	0.5	6.1	2.0
pucker outliers	4.2	3.5	7.6	7.6	3.8	5.8	2.8	0.9	12.4	9.7	6.6	9.1
suite outliers	20.4	11.3	24.2	16.7	13.5	9.6	19.4	8.3	28.5	22.0	37.2	28.6
average suiteness	0.447	0.621	0.449	0.534	0.537	0.534	0.456	0.507	0.480	0.513	0.370	0.433
R-work	0.203	0.208	0.239	0.232	0.222	0.190	0.200	0.218	0.222	0.209	0.282	0.245
R-free	0.238	0.244	0.271	0.245	0.259	0.225	0.243	0.247	0.292	0.253	0.325	0.275
RMS vs deposited		0.24		0.42		0.52		0.36		0.46		0.43

## Data Availability

Not applicable.
